# Increased Pain Sensitivity in Obese Patients After Lung Cancer Surgery

**DOI:** 10.3389/fphar.2019.00626

**Published:** 2019-06-14

**Authors:** Maciej Majchrzak, Anna Brzecka, Cyryl Daroszewski, Piotr Błasiak, Adam Rzechonek, Vadim V. Tarasov, Vladimir N. Chubarev, Anastasiya S. Kurinnaya, Tatiana I. Melnikova, Alfiya Makhmutova, Sergey G. Klochkov, Siva G. Somasundaram, Cecil E. Kirkland, Gjumrakch Aliev

**Affiliations:** ^1^Department of Thoracic Surgery, Medical University in Wroclaw, Wroclaw, Poland; ^2^Department of Pulmonology and Lung Cancer, Medical University in Wroclaw, Wroclaw, Poland; ^3^Department of Pharmacology and Pharmacy, I.M. Sechenov First Moscow State Medical University (Sechenov University), Moscow, Russia; ^4^Institute of Physiologically Active Compounds, Russian Academy of Sciences, Chernogolovka, Russia; ^5^Department of Biological Sciences, Salem University, Salem, WV, United States; ^6^GALLY International Biomedical Research Institute, San Antonio, TX, United States

**Keywords:** thoracotomy, video-assisted thoracic surgery, visual analog scale, analgesics, lung cancer, pain, obesity

## Abstract

**Background:** Obesity and cancer are recognized worldwide health threats. While there is no reported causal relationship, the increasing frequency of both conditions results in a higher incidence of obese patients who are being treated for cancer. Physiological data indicate that there is a relationship between obesity and susceptibility to pain; however, currently, there are no specific pharmacological interventions.

**Objective:** To evaluate the self-reported intensity of postoperative pain in obese and nonobese lung cancer who receive either thoracotomy or video-assisted thoracic surgery (VATS) surgical therapy.

**Material and Methods:** In 50 obese [mean body mass index (BMI) of 34.1 ± 3.2 kg/m^2^] and 62 nonobese (mean BMI of 24.9 ± 3 kg/m^2^) lung cancer patients, the intensity of pain was estimated every 4 h using a visual analog scale (VAS, 0 indicating no pain and 10 indicating “worst imaginable pain”) beginning shortly after surgery (Day O) and continuing until the day of discharge (Day D).

**Results:** The self-reported pain was more severe in obese than in nonobese patients, both at the time of the operation [Day O (4.5 ± 1.2 *vs* 3.4 ± 1.1; *p* < 0.0001)] and at the day of discharge [Day D (3.9 ± 1.4 *vs* 2.6 ± 0.9, *p* < 0.0001)]. This finding was consistent both in the patients after thoracotomy and after video-assisted thoracic surgery (VATS, *p* < 0.0001). The patients with severe pain shortly after surgery (VAS score >4) had significantly higher BMI (31.8 ± 5.6 kg/m^2^ *vs* 28.8 ± 5.2 kg/m^2^, *p* < 0.01) and were hospitalized longer than the remaining patients (13.0 ± 13.6 days *vs* 9.5 ± 3.6 days, *p* < 0.05).

**Conclusion:** The reported perception of pain in obese lung cancer patients is greater than in nonobese patients undergoing the same thoracic surgery. In obese patients, severe pain persisted longer. Pain management is an important consideration in the postoperative care of lung cancer patients, even more so with obese patients.

## Introduction

Lung cancer is the most common cancer worldwide with 1.8 million new cases in 2012 and the highest incidence rates in Central and Eastern Europe (53.5 per 100,000). These rates create significant clinical, economic, and social problems. For example, more than 1.6 million people died due to lung cancer in 2012, and this total is expected to grow to 3 million by 2035 (Ferlay et al., 2015). Obesity is another worldwide health issue. In 2014, 11% of adult men and 15% of adult women were obese. That rate is almost double that of 35 years ago (WHO, 2014). Although obesity is not an established risk factor for lung cancer (Rivera et al., 2015), the coexistence of lung cancer and obesity is commonplace due to the high baseline frequency of both (Faeh et al., 2018). In about 15–20% of patients with non-small cell lung cancer, surgical treatment is possible (Skrzypski et al., 2014, Jones et al., 2015). For most lung cancer patients, surgical treatments are possible given timely diagnostic examinations and the availability of surgical care (Royal College of Physicians, 2019).

Patients after thoracic surgical procedures are at very high risk of postoperative pain (Borys et al., 2018). Studies on the relationship between obesity and pain remain controversial (Guneli et al., 2010). Pain perception, often called the pain threshold, is reported to differ between obese and nonobese persons; however, the results are not unidirectional: both increased and decreased pain thresholds have been observed in obese patients as compared with nonobese patients (Torensma et al., 2016). Similarly, others have reported that there may be a bidirectional relationship between chronic pain and obesity (Okifuji et al., 2010, Janke and Kozak, 2012, Cooper et al., 2018). The patients with intense chronic pain more frequently suffer from eating disorders that are associated with obesity, such as nocturnal eating or excessive food intake in response to emotional situation (Godfrey et al., 2018). In some patients, chronic pain leads to increased food intake, and in obese patients, this happens three times more frequently than in nonobese patients (Bigand and Wilson, 2019).

There is also close relation between increasing weight and more frequent occurrence of chronic pain. In morbidly obese persons, chronic pain occurs four times more frequently than in nonobese persons (Hitt et al., 2007). In a recent study, obesity appeared to be one of the predictors of different types of chronic pain, such as neck pain, low back pain, and frequent headaches (Jiménez-Trujillo et al., 2019). Related to obesity factors, mechanical disturbances, chemical mediators, depression, sleep disorders, and lifestyle may all be associated with negative influence of weight excess on chronic pain (Okifuji and Hare, 2015).

Therefore, it is important to assess how obesity affects the severity of postoperative pain in patients after lung cancer surgery. A careful review of the literature related to reported pain in obese and nonobese lung cancer patients undergoing surgical procedures found no studies. Thus, the aim of the study was to provide an initial evaluation of pain experienced by obese and nonobese lung cancer patients from shortly after surgery until discharge. A collateral question was current pain management practices that include medications to reduce pain.

## Materials, Sample, and Methods

A convenience sample of 112 patients (49 women, 63 men), aged 40–84 years (mean 65 ± 6.9 years), was evaluated. Their body mass index (BMI) ranged from 15.6 to 42.9 kg/m^2^ with a mean of 29.5 ± 5.83 kg/m^2^. There were 50 obese patients (44.6%; BMI > 30 kg/m^2^) and 62 nonobese patients (55.4%), including 30 overweight patients (26.8%; BMI 25–29.9 kg/m^2^), 30 normal weight patients (26.8%; BMI 20–24.9 kg/m^2^), and 2 underweight patients (1.8%; BMI < 20 kg/m^2^). There were 41 patients (36.6%) with adenocarcinoma, 40 patients (35.7%) with squamous cell carcinoma, 16 patients (14.3%) with lung metastases, 9 patients (8%) with large cell carcinoma, 3 patients (2.7%) with adenosquamous carcinoma, and 3 patients (2.7%) with carcinoid.

Thoracotomy was performed in 55 patients (49.1%), including pulmonectomy in 5 patients, lobectomy in 34 patients, and wedge resection in 17 patients. Video-assisted thoracic surgery (VATS) was performed in 57 patients (50.9%), including lobectomy in 53 patients and wedge resection in 4 patients. There was no significant difference between thoracotomy and VATS groups regarding BMI (29.6 ± 5.3 kg/m^2^ and 28.4 ± 5.7 kg/m^2^).

The routine analgesic treatment after surgery followed detailed provided by hospital and national guidelines (Misiołek et al., 2014). It was modified individually based on the pain intensity evaluated by the patients using the visual analog scale (VAS), which was scored from 0 (no pain) to 10 (unimaginable pain). In all patients, fentanyl was administered intravenously during surgery at a dose of 0.05 mg/kg. Additionally, bupivacaine infusion paravertebrally at a concentration of 0.25% (4 ml/h) was started and continued after surgery as long as the chest drain remained inserted. After the operation, 1% morphine was administered intravenously at a rate of 1 ml/h along with intravenous ketoprofen 100 mg every 12 h and pyralgin (or paracetamol) intravenously 1 g every 6 h.

If the VAS pain rating was more than 4, the flow of morphine was increased to 2 ml/h and bupivacaine to 8 ml/h. If the pain rating did not exceed 2, then the doses of morphine were reduced to 0.5 ml/h and of bupivacaine to 2 ml/h for. If the pain rating fell between 3 and 5, then there were individual modifications using ketoprofen and pyralgin or paracetamol.

After removal of the paravertebral catheter, intravenous morphine was replaced by subcutaneously administered doses of 5 mg every 4 h; the treatment with ketoprofen and pyralgin or paracetamol was continued. The evaluation of the pain was performed by two scorers independently: by a nurse shortly after surgery and by a doctor at the time of discharge. At Day D, the scorer was unaware of the score at Day O. The results were compared in the groups of obese (BMI ≥ 30 kg/m^2^) and nonobese (BMI < 30 kg/m^2^) patients.

Statistical analyses were performed with the statistical version (StatSoft, Inc). 12. The normality of variables was tested with Kolmogorov–Smirnov test. Data were compared using the Student’s *t* test. Mean with standard deviations were used, and an alpha significance level of *p* < 0.05 was accepted.

## Results

The comparison of the groups of the obese and nonobese patients after surgical procedures for lung cancer is shown in **Table 1**. There were similar percentages of men and women in both groups. Patients in both groups were of similar age and height. The differences in weight and BMI were statistically significant, as expected. Within the obese group, there were no statistically significant differences based on who underwent thoracotomy or VATS.

**Table 1:** The comparison of the groups of the obese and nonobese lung cancer patients who underwent thoracotomy or video-assisted thoracic surgery (VATS).

	Nonobese patients (*n* = 62)	Obese patients (*n* = 50)	*p*
Women (%)	43.5	44	ns
Men (%)	56.5	56	ns
Age (years)	64.2 ± 8	65.6 ± 5.7	ns
Height (cm)	165.6 ± 9.3	166.4 ± 9	ns
Weight (kg)	68.5 ± 11.7	94.6 ± 13.1	<0.0001
BMI (kg/m^2^)	24.9 ± 3	34.1 ± 3.2	<0.0001
Patients after thoracotomy (%)	42	58	ns
Patients after VATS (%)	58	42	ns

ns, not statistically significant; BMI, body mass index.

The length of stay in hospital ranged from 4 to 78 days. The mean time of hospitalization was similar in obese and nonobese patients (**Table 2**). The differences in the length of stay were not significant in the groups of obese, overweight, and normal weight/underweight patients (**Table 3**).

**Table 2:** Length of stay in hospital and intensity of pain in the surgically treated obese and nonobese lung cancer patients.

	Nonobese patients (*n* = 62)	Obese patients (*n* = 50)	*p*
Length of stay in hospital (days)	10.3 ± 4.7	10.6 ± 10.3	ns
Intensity of pain, Day O	3.4 ± 1.1	4.5 ± 1.2	<0.0001
Intensity of pain, Day D	2.6 ± 0.9	3.9 ± 1.4	<0.0001

**Table 3:** Comparison of the length of stay in hospital and the intensity of pain in surgically treated lung cancer patient groups of obese (I), overweight (II), and normal weight/underweight patients (III).

	I Obese patients (*n* = 50)	II Overweight patients (*n* = 30)	III Normal weight and underweight patients (*n* = 32)	*p* I vs II	*p* II vs III	*p* I vs III
Length of stay in hospital (days)	10.6 ± 10.3	11.0 ± 5.3	9.6 ± 3.9	ns	ns	ns
Intensity of pain, Day O	4.5 ± 1.2	3.7 ± 1.1	3.1 ± 1.1	<0.01	<0.05	<0.0001
Intensity of pain, Day D	3.9 ± 1.4	2.9 ± 0.9	2.4 ± 0.9	<0.001	<0.05	<0.0001

In all the patients, the intensity of pain ranged from 0 to 8 both at Day O and at Day D, and its mean intensity was 3.9 ± 1.3 at Day O and 3.2 ± 1.3 at the time of leaving hospital, Day D. In the whole group, there were no significant differences in the pain scores between the values recorded in the morning/early afternoon hours (6.00–14.00) and afternoon/night hours (18.00–02.00) in the obese, overweight, and normal weight patients.

There were 29 patients (26%) who suffered from severe pain (score >4) at day O. The patients suffering from severe pain, scored as exceeding 4, shortly after surgery as compared with the remaining patients had higher BMI (31.8 ± 5.6 kg/m^2^ *vs* 28.8 ± 5.2, *p* < 0.01) and stayed longer in the hospital (13.0 ± 13.6 days *vs* 9.5 ± 3.6 days, *p* < 0.05). Patients with intensity of pain exceeding 4 at Day O (mean 5.6 ± 0.9) still suffered from severe pain at Day D (mean 4.9 ± 1.2).

The pain was significantly more intense in obese than in nonobese patients, both shortly after surgery and at the time of discharge home (**Table 2**).

The intensity of pain—both shortly after surgery and at the time of discharge from hospital—in the obese patients (mean BMI 34.1 ± 3.2 kg/m^2^) was greater than in the overweight patients (mean BMI 27.4 ± 1.6 kg/m^2^), and the intensity of pain in overweight patients was greater than in the normal weight and underweight patients (mean BMI 22.6 ± 2 kg/m^2^); the data is shown in **Table 3**.

Taking into account the slight differences in the frequency of thoracotomy and VATS performed in the obese and nonobese groups, the intensity of pain was compared separately in the patients who underwent thoracotomy and VATS (**Table 4**). The pain was more intense both on Day O and Day D for the obese compared to nonobese patients, regardless of the procedure: open thoracotomy or VATS.

**Table 4:** The intensity of pain comparison in the groups of obese and nonobese lung cancer patients who underwent thoracotomy and VATS.

Thoracotomy	Nonobese patients (*n* = 27)	Obese patients (*n* = 29)	*p*
VATS	Nonobese patients (*n* = 35)	Obese patients (*n* = 21)	*p*
Intensity of pain, Day O	3.9 ± 1.3	4.9 ± 1.3	<0.01
Intensity of pain, Day D	3.1 ± 1.1	4.5 ± 1.4	<0.001
Intensity of pain, Day O	3.0 ± 0.8	4.0 ± 0.8	<0.0001
Intensity of pain, Day D	2.3 ± 0.6	3.1 ± 0.8	<0.0001

Shortly after surgery (Day O), the most intense pain was in the group of obese patients who underwent thoracotomy (4.9 ± 1.3) and the slightest in the nonobese patients who underwent VATS (3.0 ± 0.8). At the time of discharge from hospital (Day D), the most intense pain was in the group of obese patients who underwent thoracotomy (4.5 ± 1.4) and the slightest in the nonobese patients who underwent VATS (2.3 ± 0.6). In both Day O and Day D, the intensity of pain in obese patients exceeded the intensity of pain in nonobese patients by about two points using the VAS.

## Discussion

The results of our study indicate that obese patients after surgical treatment of lung cancer suffer more from pain in the postoperative period than nonobese patients, both Day O and Day D. This finding is independent of the type of surgery, either thoracotomy or VATS.

Similar results were observed in some, but not all, other studies comparing pain sensitivity after various surgical procedures (Rashiq and Dick, 2014). More severe early postoperative pain in obese as compared with nonobese patients recently was reported after total joint arthroplasty (Campbell et al., 2018). After orthodontic treatment with fixed appliances placement, the orofacial pain was more severe during first week after procedure in obese than in nonobese patients (Saloom et al., 2018). Obesity was related to more severe pain in the first month after mastectomy (Fecho et al., 2009) and to more frequent occurrence of neuropathic pain after mastectomy (Smith et al., 1999). Obesity appeared to be a factor of long-lasting postoperative pain in adolescents who were operated on for scoliosis, as indicated by prolonged opioid use (Yang and Werner, 2018).

However, in the patients undergoing total hip arthroplasty, obesity was not associated with increased postoperative pain (Motaghedi et al., 2014). Retrospective analysis of opioid consumption in the postoperative period in the patients undergoing surgical procedures for degenerative spinal pathology revealed no differences between obese and nonobese patients (Narain et al., 2018).

There are also a number of studies on the relation between obesity and non-postoperative, chronic pain, indicating that obese patients have lower pain thresholds (Stone and Broderick, 2012). Obesity was independently associated with chronic pain, as recently shown in the study of 4,993 persons with multivariable odds ratios 2.09 (calculated with 95% confidence intervals 1.20–3.640) (Yamada et al., 2018). Obesity appeared to be a factor associated with neuropathic pain, independent from musculoskeletal causes of chronic pain, such as osteoarthritis or low back pain (Hozumi et al., 2016). In the patients with painful diabetic polyneuropathy, obesity was related to more severe pain (Spallone et al., 2011). Decreased pain threshold was found in obese as compared with nonobese patients with fibromyalgia (Neumann et al., 2008; Okifuji et al., 2010; Kim et al., 2012). Obese women with fibromyalgia suffered more from chronic pain than nonobese fibromyalgia women (Koçyiğit and Okyay, 2018).

However, experimental data gave contradictory results. In experiments in animals, pain induced by thermal stimulus indicated increase in pain threshold related to diet-induced obesity (Ramzan et al., 1993). Pain induced by pressure on the skin was less intense in obese than nonobese persons (Khimich, 1997). In the other study, lower pain thresholds in obese than in nonobese persons were found in response to pressure stimulus, but not to thermal stimulus (Tashani et al., 2017). Electrical stimulation used to evoke pain revealed both increased (Zahorska-Markiewicz et al., 1983; Miscio et al., 2005; Dodet et al., 2013) and decreased (Pradalier et al., 1982) pain threshold in obese people as compared with nonobese persons.

The reason of different pain thresholds in the obese and nonobese patients has not been elucidated. Chronic inflammatory state is associated with obesity (Divella et al., 2016) and thus may partially explain the relationship between excess weight and pain.

It has been reported that the systemic inflammation due to the release of inflammatory mediators from macrophage accumulation is greater in obese than in nonobese patients. This might contribute to lower thresholds of pain in obese patients (Weisberg et al., 2003).

Recently, among adipokines (i.e., cytokines associated with chronic inflammatory state and obesity) such as leptin, adiponectin, and resistin, only the serum level of resistin was found to be related strongly to the severity of pain in the patients undergoing laparotomy (Hozumi et al., 2018). In another recent study, obesity was found to be linked with genetic polymorphism altering sensitivity to pain, thus indicating that chronic pain might be related both to obesity and genetic factors (Elmallah et al., 2018).

The authors of this paper speculate that one of the reasons of decreased pain threshold in obese patients might be nocturnal hypoxemia: There is a high percentage of sleep apnea among these obese patients, often undiagnosed and untreated. It has been shown that nocturnal episodes of arterial oxygen desaturations as a consequence of obstructive sleep apneas and hypopneas are associated with an increased pain (Doufas et al., 2013). The problem of the relation between chronic recurrent hypoxemia and increased pain sensitivity needs further evaluation.

More than one quarter of our patients (26%) suffered from severe pain (score exceeding 4 on VAS) in the post-general anesthesia unit. These patients had higher BMI than the patients with less intense pain (31.8 ± 5.6 kg/m^2^ *vs* 28.8 ± 5.2, *p* < 0.01). The proportion of patients suffering from severe pain requiring interventional analgesics pain varies depending on the groups of patients. One large-scale retrospective study encompassing 1,736 patients found that, for 28.5% of patients in the study, obesity was an independent factor of postoperative pain requiring intervention (odds ratio 1.683; 90% CI 1.226–2.310; *p* = 0.001) (Mei et al., 2010).

All our patients received the best analgesic treatment according to guidelines supervised mostly by nurses and modified, when needed, by doctors. The problem of unsatisfactory analgesic treatment in postsurgical period remains unresolved in other centers (Mędrzycka-Dąbrowska et al., 2016; Borys et al., 2018; Tomaszek and Dębska, 2018).

In our patients, high ratings of pain intensity Day O and Day D are noted. This finding confirms individual differences in pain perception.

The length of stay of our patients in hospital after surgery for lung cancer was similar in obese and nonobese patients. This is in accordance with a recent study of lung cancer obese and nonobese patients who underwent lobectomy (Wang et al., 2018). However, the length of stay of the patients with severe pain early in the postoperative period (who also had higher BMI) was significantly longer than the patients who suffered less in the postgeneral anesthesia room.

There were significant differences in the pain sensation in obese and nonobese persons in the group of patients who underwent open thoracotomy or VATS, confirming our finding of close relation of pain sensitivity and increased BMI. Possible differences in pain intensity in the patients after VATS and open thoracotomy should be mentioned. In general, VATS is known as a procedure associated with less severe pain than open thoracotomy (Bendixen et al., 2016, Fang et al., 2018). However, recently, in patients operated for lung cancer, no differences in postoperative pain score were found between VATS and open thoracotomy (van der Ploeg et al., 2019).

The strength of the study is appropriate number of the patients studied in compared groups. The other one is double check of pain intensity soon after surgical procedure and at the time of discharge home.

The main limitation of this study is a use of only one scale for determination of pain intensity in the postoperative period. However, the scale used, VAS, is routinely performed scale for evaluation of pain in surgical unit of our Hospital, and both the nurses and the doctors are familiar with it. Neuropathic pain occurs usually with a delay of 7 days after thoracic surgery and persists thereafter (Homma et al., 2018); thus, the scales for neuropathic pain could be used at the end of the stay in hospital as a mean duration of hospitalization of our patients was 10.4 ± 7.7 days. However, this would not allow to repeat the evaluation of the pain intensity, as the study was not extended to ambulatory care of the patients.

## Future Directions of the Research

It would be interesting to continue the evaluation of the delayed, mostly neuropathic, pain in the obese and nonobese groups of the patients with the use of the scales for delayed postoperative pain.

Taking into account that obese persons are at high risk of developing hypoxemia or hypoventilation during sleep due to obstructive sleep apnea syndrome (Brzecka and Davies, 1993) and that opioids—routinely used for analgesia after thoracic surgery—may independently lead to or aggravate sleep apneas and sleep hypoventilation (Macintyre et al., 2011), further studies on the risk of respiratory insufficiency during sleep in obese patients after open thoracotomy or VATS are needed.

## Conclusion

Obese patients undergoing surgery for lung cancer suffer more from the pain during the postoperative period than nonobese patients, and this observation may indicate higher sensitivity to pain in the patients with increased BMI. Severe pain in early post-surgical period is associated with a high probability of persisting severe pain at the time of discharge. There should be more rigorous supervision of analgesic treatment in postoperative period in the obese patients with lung cancer in order to prevent the patients’ suffering from severe pain.

## Ethics Statement

The routine analgesic treatment after surgery followed detailed guidelines in our hospital and according to national and international guidelines (Misiołek et al., 2014) and was modified individually based on the pain intensity evaluated by the patients with the use of visual analog scale (VAS) and scored from 0 to 10. For the detail please see manuscript file.

## Author Contributions

MM, AB, CD, PB, and AR performed data acquisition, organization of the database, and statistical analysis. CD, AB, PB, AR, VVT, VNC, ASK, TIM, AM, SGK, SGS, CEK, and GA contributed to the design of the study, conception of the study, and writing of the manuscript. GA, CEK, SGS, and MM prepared the preparation and editing of the manuscript. All authors contributed to manuscript revisions and approved the submitted version.

## Funding

This work was supported by Russian Academic Excellence project “5-100” for the Sechnov University, Moscow, Russia. This research was partially also supported in part within the framework of the State Proposal 0090-2017-0018, and by the project of RAS Program “Fundamental Research for Biomedical Technologies’ (IPAC, RAS Chernogolovka, Moscow Region, Russia).

## Conflict of Interest Statement

GA was employed by GALLY International Biomedical Research Consulting LLC, San Antonio, Texas, USA.

The remaining authors declare that the research was conducted in the absence of any commercial or financial relationships that could be construed as a potential conflict of interest.
